# Bisphenol A, Bisphenol F, and Bisphenol S: The Bad and the Ugly. Where Is the Good?

**DOI:** 10.3390/life11040314

**Published:** 2021-04-03

**Authors:** Sophie Fouyet, Elodie Olivier, Pascale Leproux, Mélody Dutot, Patrice Rat

**Affiliations:** 1 UMR CNRS 8038, Laboratoire de Chimie-Toxicologie Analytique et Cellulaire, Faculté de Pharmacie de Paris, Université de Paris, 75006 Paris, France; elodie.olivier@u-paris.fr (E.O.); pascale.leproux@u-paris.fr (P.L.); melody.dutot@yslab.fr (M.D.); patrice.rat@u-paris.fr (P.R.); 2 Recherche & Développement, Yslab, 29000 Quimper, France

**Keywords:** human placental toxicity, bisphenol A, bisphenol F, bisphenol S, P2X7 receptor, inflammasome, apoptosis

## Abstract

Background: Bisphenol A (BPA), a reprotoxic and endocrine-disrupting chemical, has been substituted by alternative bisphenols such as bisphenol F (BPF) and bisphenol S (BPS) in the plastic industry. Despite their detection in placenta and amniotic fluids, the effects of bisphenols on human placental cells have not been characterized. Our objective was to explore in vitro and to compare the toxicity of BPA to its substitutes BPF and BPS to highlight their potential risks for placenta and then pregnancy. Methods: Human placenta cells (JEG-Tox cells) were incubated with BPA, BPF, and BPS for 72 h. Cell viability, cell death, and degenerative P2X7 receptor and caspases activation, and chromatin condensation were assessed using microplate cytometry and fluorescence microscopy. Results: Incubation with BPA, BPF, or BPS was associated with P2X7 receptor activation and chromatin condensation. BPA and BPF induced more caspase-1, caspase-9, and caspase-3 activation than BPS. Only BPF enhanced caspase-8 activity. Conclusions: BPA, BPF, and BPS are all toxic to human placental cells, with the P2X7 receptor being a common key element. BPA substitution by BPF and BPS does not appear to be a safe alternative for human health, particularly for pregnant women and their fetuses.

## 1. Introduction

**Figure 1:**
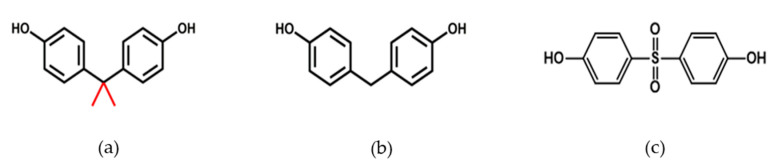
Chemical structure of: (**a**) BPA, (**b**) BPF, and (**c**) BPS.

Bisphenol A (BPA; 4,4′-(propane-2,2-diyl)diphenol) is a phenolic compound discovered in the late nineteenth century, used in the manufacturing of polycarbonates, epoxyresins, and other polymers. It is therefore found in a wide range of products such as food and beverage containers, compact discs, personal protective equipment, sport equipment, and medical equipment, leading to multiple sources of exposure for the entire population. In humans, BPA is detected in the blood and urine, but it is also found in the placenta and amniotic fluid [1,2,3]. BPA has been listed as a substance of very high concern (SVHC) under the registration, evaluation, authorization and restriction of chemicals (REACh) legislation, first because of its reprotoxic properties and then because of its endocrine-disrupting properties. Its use has been limited and banned in baby bottles in Canada (2008), France (2010), and European Union (2011). In France, since January 2015, BPA has been forbidden in food and beverage packaging. These restrictions led manufacturers to use alternative bisphenols such as bisphenol F (BPF; bis(4-hydroxyphenyl)methane); Figure 1b) and bisphenol S (BPS; bis[4-hydroxyphenyl]sulfone; Figure 1c). According to the European Agency (ECHA), 187 kilotons of BPS-based thermal paper were placed on the EU market in 2019. By 2022, it is expected that 61% (or 307 kilotons) of all thermal paper in the EU will be BPS-based. However, despite the increasing use of BPA structural analogs, there is limited information on the potential placental and fetal toxicity of these molecules.

The placenta is a crucial organ for pregnancy, acting as an endocrine organ and being an interface between the mother and the fetus. Consequently, it plays a central role in the health of the fetus, and has a lifelong impact on their future wellbeing. Disordered placental development is the primary defect in the majority of pregnancy diseases, such as preeclampsia (considered the leading cause of maternal and fetal death worldwide), fetal growth restriction, recurrent miscarriage, and stillbirth [4,5,6,7,8,9,10]. The link between preeclampsia and BPA exposure has not been clearly established yet, but concentrations of BPA are higher in women suffering from preeclampsia [3,11]. It has also been published that BPA affects the placenta in mice and therefore alters fetal programming [12]. Benachour et al. showed in vitro that even very low concentrations of BPA are able to induce apoptosis, necrosis, and inflammation in human trophoblastic cells [13]. The question that we raise is: Could BPA analogs also be at the origin of preeclampsia through the induction of trophoblast death? At the cell level, it has been suggested that preterm birth [14] and preeclampsia [15] are triggered by P2X7 receptor activation. The activation of the P2X7 receptor, expressed by placental cells [16], leads to different pathways that are implicated in preeclampsia, including apoptosis and necrosis [17], and inflammation with NLRP3 inflammasome and caspase-1 activation [18]. In light of the above, the P2X7 receptor seems to be a good marker of placental toxicity.

Our aim was to compare bisphenol A toxicity to its substitutes, bisphenol F and bisphenol S, on their ability to induce in vitro cell death in human placental cells, at concentrations that can be found in the placenta, in order to highlight the potential risks for the placenta and then pregnancy.

## 2. Materials and Methods

Chemicals and reagents. Cell culture reagents: Minimum essential medium (MEM), fetal bovine serum (FBS), 2 mM glutamine, 100 U/mL of penicillin, 100 μg/mL of streptomycin, trypsin-EDTA 0.05%, and phosphate-buffered saline (PBS) were provided by Gibco (Paisley, U.K.), and cell culture material such as flasks and microplates by Corning (Schiphol-Rijk, The Netherlands). YO-PRO-1^®^, CellEvent^TM^ Caspase-3/7 Green Detection Reagent and Hoechst 33342 probes were obtained from ThermoFisher Scientific (Waltham, MA, USA), while Caspase-Glo^®^ 1 Inflammasome Assay, Caspase-Glo^®^ 8 Assay, and Caspase-Glo^®^ 9 Assay were from Promega (Madison, WI, USA). All chemicals were purchased from Sigma-Aldrich (Saint Quentin Fallavier, France).

Bisphenol A, bisphenol F, and bisphenol S were dissolved in dimethylsulfoxyde (DMSO). Stock solutions were stored at −20 °C and work solutions were obtained after a 1/1000 dilution in culture medium. The final concentration of DMSO on cells was less than or equal to 0.1%.

Human placental cell culture. JEG-3 cells were selected in our study for several reasons. Primary cultures of human placental cells, in addition to being difficult to obtain and maintain in culture, may have been exposed to BPA, BPF, and BPS or other chemicals during pregnancy, which would lead to bias in data analysis. Several human placental cell lines are available in international collections such as the American Type Culture Collection (ATCC). Previous studies have shown that the JEG-3 cell line provides an appropriate model to detect placental toxicity [19,20]. JEG-3 cells are furthermore able to synthesize and secrete hormones [20,21]. For BPA, being an endocrine disrupting chemical, JEG-3 cells are the best option to compare human placental toxicity of bisphenols.

The JEG-3 human trophoblast cell line was obtained from ATCC (HTB-36). Cells were cultured in minimum essential medium (MEM) supplemented with 10% fetal bovine serum (FBS), 1% L-glutamine, and 0.5% penicillin and streptomycin in 75 cm^2^ polystyrene flasks. Cell cultures were maintained in a cell culture incubator (37 °C, saturated humidity, and 5% CO_2_). When the JEG-3 cells reached subconfluency, they were detached using trypsin-EDTA and counted. The cellular suspension was diluted and seeded in 96-well microplates at a cellular density of 80,000 cells/mL (200 µL/well), then kept at 37 °C for 24 h. The cells were incubated for 72 h with BPA, BPF, and BPS in MEM supplemented with 2.5% FBS according to Olivier *et al.*’s protocol that describes the JEG-Tox model [20].

Cell viability: Neutral Red assay. The Neutral Red solution at 0.4% (m/v in water) was diluted in cell culture medium to obtain a working concentration of 50 μg/mL. Neutral Red working solution was distributed in the plates for a 3 h incubation time at 37 °C. The cells were then rinsed with PBS and lysed with a solution of ethanol–water–acetic acid (50.6/48.4/1, v/v/v). After homogenization, the fluorescence signal was scanned (λ_exc_ = 540 nm and λ_em_ = 600 nm) using a Spark^®^ microplate reader (Tecan, Männedorf, Switzerland).

P2X7 receptor expression: Western blot technique. Cell lysates were prepared by washing the cells twice in cold PBS, lysing them in RIPA buffer (Sigma-Aldrich) supplemented with protease inhibitor cocktail (Sigma-Aldrich) for 15 min on ice, and then, the obtained lysates were sonicated for 30 s. The total protein concentration was determined using the Pierce^®^ microBCA Protein Assay Kit (Thermofisher Scientific). Protein sample buffer and reducing agent were added and the samples were boiled at 70 °C for 10 min. Then, 40 μg of these samples was loaded and electrophoretically separated on 4–12% polyacrylamide gels (BoltTM Bis-Tris Plus, Thermofisher Scientific), before being transferred to nitrocellulose membrane (iBlot™ 2 Transfer Stack, 0.2 μm Nitrocellulose, ThermoFisher Scientific) using iBlot™ 2 Gel transfer device (ThermoFisher Scientific). Blots were incubated first using a primary polyclonal rabbit anti-P2X7 at 1:1000 (overnight, catalog #PA5-25581, ThermoFisher Scientific) and then a secondary DyLight 800 Fluor-conjugated anti-rabbit antibody at 1:15,000 for 1 h (ThermoFisher Scientific). Actin was used as a housekeeping protein for the normalization of data using an anti-actin polyclonal antibody at 1:1000 (catalog #PA5-11570, ThermoFisher Scientific). Blots were scanned using Odyssey^®^ Imaging System (Li-COR Biosciences, Lincoln, NE, USA). Quantitation of bands was performed using ImageJ software.

Cell death P2X7 receptor activation: YO-PRO-1^®^ assay. P2X7 cell death receptor activation was evaluated using the YO-PRO-1^®^ assay [22]. YO-PRO-1^®^ probe only enters into cells after pore opening induced by P2X7 receptor activation, and binds to DNA, emitting fluorescence. A 1 mM YO-PRO-1^®^ stock solution was diluted at 1/500 in PBS just before use and distributed in the wells of the microplate. After a 10 min incubation time at room temperature, the fluorescence signal was read (λ_ex_ = 485 nm and λ_em_ = 531 nm) using a Spark^®^ microplate reader.

Caspase-1, -8, and -9 activity: Caspase-Glo^®^ Assays. Caspase-1, -8, and -9 activity was evaluated using the Caspase-Glo^®^ 1, 8, and 9 Assay Kits, respectively. The assay was performed according to the manufacturer’s instructions. Luminescence was quantified using a Spark^®^ microplate reader.

Caspase 3 activity: CellEvent^TM^ Caspase-3/7 Green Detection Reagent. Caspase-3 activity was evaluated using the CellEvent^TM^ Caspase3/7 Green Detection Reagent. Cell Event^TM^ Caspase-3/7 Green Detection reagent was diluted in PBS with 2.5% FBS to a final concentration of 8 μM. The cells were incubated with the reagent for 30 min and then rinsed with PBS. The cells were observed under fluorescence microscopy and pictures were taken (Evos FL, Thermo Fisher Scientific, Waltham, MA, USA).

Quantitative and qualitative analysis of chromatin condensation: Hoechst 33342 assay. Chromatin condensation was evaluated using the Hoechst 33342 assay (ThermoFisher Scientific, Illkirch, France). Hoechst 33342 fluorescent probe enters and intercalates into DNA in living and apoptotic cells. The fluorescent signal is proportional to chromatin condensation, and the nuclei of living cells are blue, while the nuclei of apoptotic cells are bright [20,23,24]. A 0.5 μg/mL Hoechst 33342 solution was distributed in the wells of the microplate. The fluorescence signal was read after a 30 min incubation time at room temperature (λ_ex_ = 350 nm and λ_em_ = 450 nm) using a Spark^®^ microplate reader, and the cells were observed under fluorescence microscopy and pictures were taken (Evos FL, Thermo Fisher Scientific).

Results exploitation and statistical analysis. The results are expressed in percentage or fold change compared to control cells and presented as means of at least three independent experiments ± standard errors of the mean.

Statistical analysis was performed using GraphPad Prism 8 software (La Jolla, CA, USA). A one-way analysis of variance for repeated measures followed by a Dunnett’s test with risk α set at 5% were performed. Thresholds of significance were **** *p* < 0.0001, *** *p* < 0.001, ** *p* < 0.01, and * *p* < 0.05 compared to control cells.

## 3. Results

### 3.1. Cell Viability

We investigated JEG-Tox cell viability after incubation with bisphenols using the Neutral Red assay. Any concentration inducing a loss of cell viability greater than or equal to 30% was considered as cytotoxic (ISO 2009). Therefore, no statistical analysis was performed.

**Figure 2:**
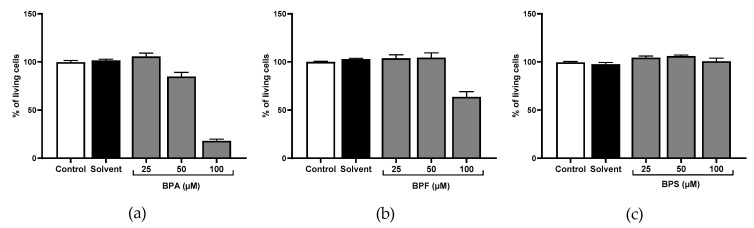
Cell viability was evaluated using the Neutral Red assay after (**a**) BPA, (**b**) BPF, and (**c**) BPS incubation for 72 h in JEG-Tox cells. Data correspond to the mean ± standard error of the mean (SEM) of four independent experiments.

No loss of cell viability was observed after 72 h with BPS up to 100 μM (Figure 2c). In contrast, BPA and BPF at 100 μM reduced cell viability to 18% and 64%, respectively (Figure 2a,b), and were thus cytotoxic at 100 μM. To evaluate subcytotoxic concentrations, BPA and BPF at 100 μM were suppressed in the rest of the study.

### 3.2. P2X7 Receptor Activation and Expression

**Figure 3:**
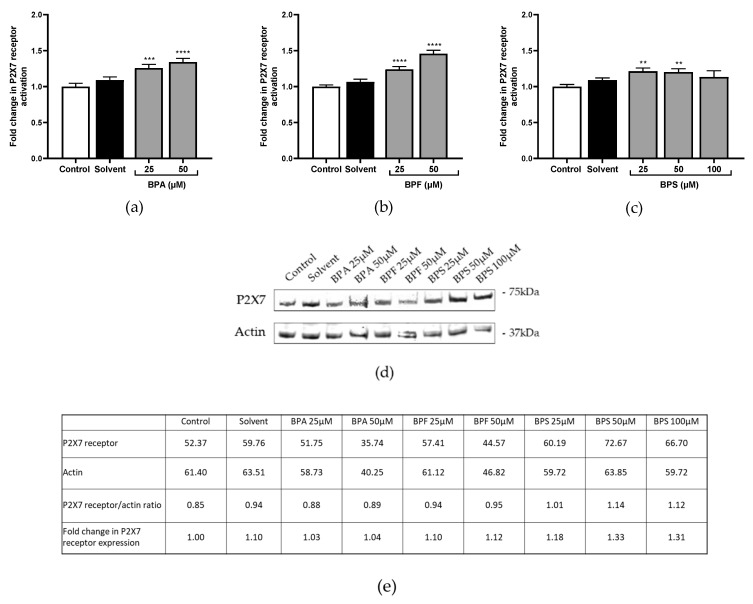
(**a**–**c**) P2X7 receptor activation was evaluated after (**a**) BPA, (**b**) BPF, and (**c**) BPS incubation for 72 h in JEG-Tox cells. Data correspond to the mean ±SEM of four independent experiments. The significance thresholds were **** *p* < 0.0001, *** *p* < 0.001, and ** *p* < 0.01 compared to the control. (**d**,**e**) P2X7 receptor expression was assessed after BPA, BPF, and BPS incubation for 72 h in JEG-Tox cells (**d**) using the Western blot technique, and (**e**) quantitative analysis was performed using ImageJ software. β-actin was used as a control.

P2X7 pore opening, reflecting P2X7 receptor activation, was assessed using the fluorescent YO-PRO-1^®^ assay. P2X7 receptor was significantly activated by the three tested bisphenols. BPA and BPF induced similar levels of P2X7 activation in a concentration-dependent manner (×1.26 at 25 μM and ×1.34 at 50 μM in Figure 3a; ×1.24 at 25 μM and ×1.46 at 50 μM compared to the control in Figure 3b). BPS induced slighter activation than the two other bisphenols (×1.21 and ×1.20 at 25 μM and 50 μM, respectively; Figure 3c).

P2X7 receptor expression was investigated after incubation with bisphenols using the Western blot technique. Only BPS enhanced P2X7 receptor expression compared to the control (×1.18 at 25 μM, ×1.33 at 50 μM, and ×1.31 at 100 μM; Figure 3d).

### 3.3. Caspase-1, Caspase-8, Caspase-9, and Caspase-3 Activity

The bioluminescent Caspase-Glo^®^ 1 inflammasome, Caspase-Glo^®^ 8, and Caspase-Glo^®^ 9 assays were used to quantify caspase-1, caspase-8, and caspase-9 activity, respectively, and the fluorescence CellEvent^®^ Caspase-3/7 Green Detection Reagent was used to analyze caspase-3 activity by microscopy.

**Figure 4:**
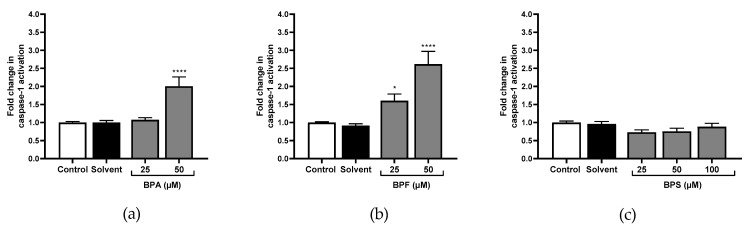
Caspase-1 activity cells was evaluated in JEG-Tox after incubation with (**a**) BPA, (**b**) BPF, and (**c**) BPS incubation for 72 h in JEG-Tox cells. Data correspond to the mean ± SEM of four independent experiments. The significance thresholds were **** *p* < 0.0001 and * *p* < 0.05 compared to the control.

**Figure 5:**
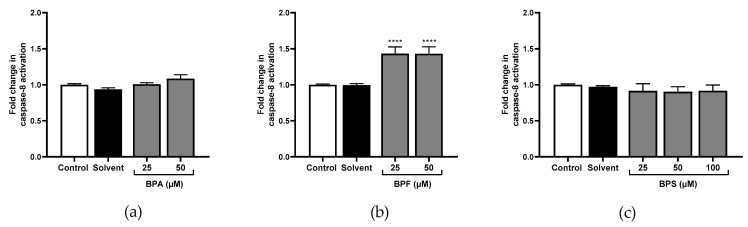
Caspase-8 activity cells was evaluated in JEG-Tox after incubation with (**a**) BPA, (**b**) BPF, and (**c**) BPS incubation for 72 h in JEG-Tox cells. Data correspond to the mean ±SEM of four independent experiments. The significance threshold was **** *p* < 0.0001compared to the control.

**Figure 6:**
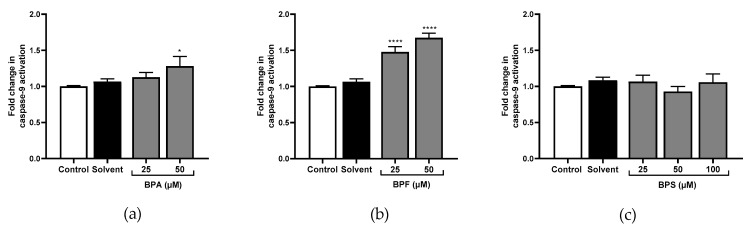
Caspase-9 activity cells was evaluated in JEG-Tox after incubation with (**a**) BPA, (**b**) BPF, and (**c**) BPS incubation for 72 h in JEG-Tox cells. Data correspond to the mean ± SEM of four independent experiments. The significance thresholds were **** *p* < 0.0001 and * *p* < 0.05 compared to the control.

**Figure 7:**
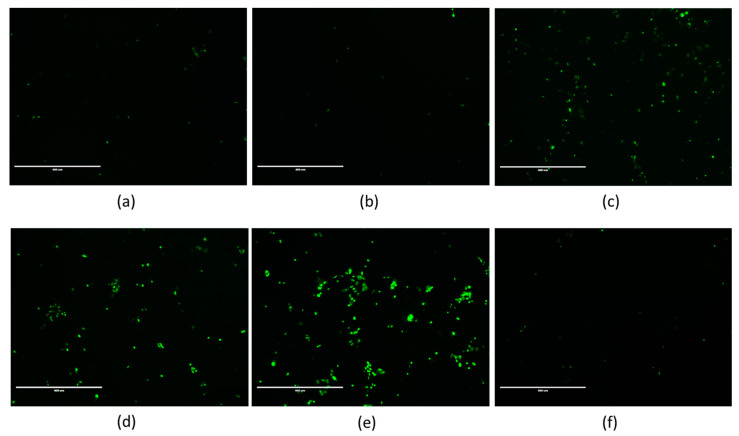
Fluorescence microscopy images of JEG-Tox cells stained for caspase-3/7 activity. After 72 h of incubation with (**a**) the control, (**b**) solvent, (**c**) 50 μM of BPA, (**d**,**e**) 25 μM and 50 μM of BPF, respectively, and (**f**) 100 μM of BPS, the cells were stained using Caspase-3/7 Green Detection Reagent. The data shown are representative of three independent experiments. The images were captured under the same acquisition parameters by Evos FL fluorescence microscope (Thermo Fisher Scientific).

Only the highest concentration of BPA significantly activated caspase-1 (×1.57 at 50 μM; Figure 4a). No effect was observed for caspase-8 (Figure 5a). However, caspase-9 (×1.28; Figure 6a), and caspase-3 (Figure 7c) were significantly activated by the highest concentration of BPA.

All tested concentrations of BPF significantly activated caspase-1 compared to the control (×1.60 and ×2.61 at 25 μM and 50 μM respectively; Figure 4b). Caspase-8 was also significantly activated at 25 μM and 50 μM of BPF (×1.43; Figure 5b), and so was caspase-9 (×1.47 and ×1.67, respectively; Figure 6b). Caspase-3 activity was higher with 25 μM and 50 μM of BPF than the control (Figure 7d,e).

BPS had no effect on caspase-1 (Figure 4c), caspase-8, or caspase-9 activities (Figure 5c and Figure 6c), nor caspase-3 activities (Figure 7f).

### 3.4. Chromatin Condensation

**Figure 8:**
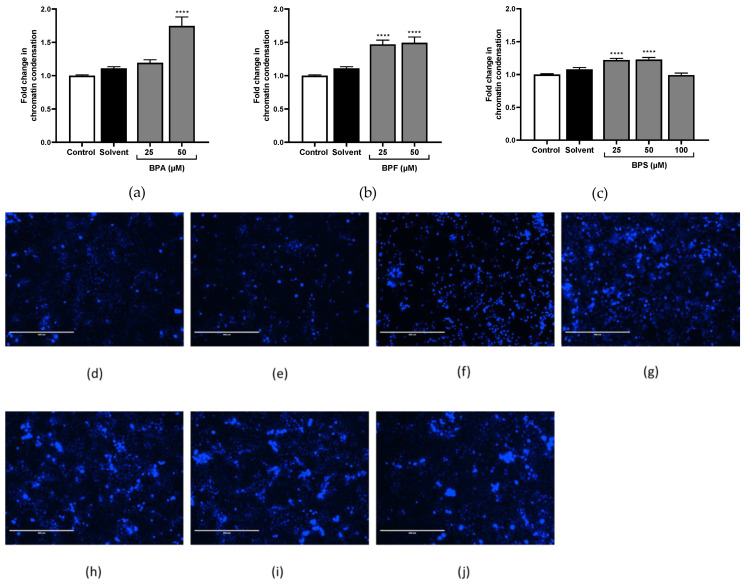
Quantification and qualification of chromatin condensation using Hoechst 33,342 assay. (**a**–**c**) Chromatin condensation quantification was assessed after (**a**) BPA, (**b**) BPF, and (**c**) BPS incubation for 72 h in JEG-Tox cells. Data correspond to the mean ± SEM of four independent experiments. The significance threshold was **** *p* < 0.0001 compared to the control. (**d**–**j**) Chromatin condensation observation under fluorescence microscopy in JEG-Tox cells after incubation for 72 h with (**d**) the control, (**e**) solvent, (**f**) 50 μM of BPA, (**g**,**h**) 25 μM and 50 μM of BPF, respectively, and (**i**,**j**) 25 μM and 50 μM of BPS, respectively. The pictures are representative of three independent experiments and were captured under the same acquisition parameters.

The three tested bisphenols condensed significantly the chromatin. BPA induced the most chromatin condensation, but only at 50 μM (×1.79; Figure 8a,f). BPF triggered chromatin condensation at two tested concentrations (×1.47 and 1.49 at 25 μM and 50 μM, respectively; Figure 8b,g,h). BPS induced slighter activation than two other bisphenols, but from 25 μM (×1.22 at 25 μM and 1.23 at 50 μM; Figure 8c,i,j).

## 4. Discussion

The objective of the present study was to explore and compare the toxicity of BPA to its substitutes BPF and BPS in a human placental cell line to highlight their potential risks for pregnancy.

In humans, BPA is detected in amniotic fluid, neonatal blood, and the placenta at levels of nanograms/mililiter [1,2,3,25,26]. During fetal development, BPA can be accumulated in the placenta and amniotic fluid; indeed, a 5-fold higher concentration was revealed in amniotic fluid than in maternal plasma [25]. BPA was reported to significantly affect fetal development and to increase the risk of adverse health consequences. Associations between BPA exposure and reproductive dysfunction [27,28], obesity [29,30], developmental behavioral problems [31,32], cancers [33,34,35], and placental disorders [3,12,36] have been reported. For all of these reasons, the use of BPA has been limited or banned in several countries, leading to its substitution by analogs, mainly BPF and BPS. Are BPF and BPS truly safe alternatives to BPA? That is a fair question, since BPF is also detected in urine, the uterus, the placenta, amniotic fluid, and fetuses [37,38,39], and BPS in urine, maternal plasma, cord plasma, and placenta samples [38,39,40,41,42], both at levels of nanograms/mililiter [38]. BPS concentrations in the placenta are significantly higher than in maternal plasma [42], suggesting BPS accumulation in the placenta, just like BPA [25,43]. The concentrations of bisphenols used in this study are of the same order of magnitude as those levels.

The present study is the first to demonstrate that BPA, BPF, and BPS are all toxic to placental cells, but with different impacts. In particular, cell viability, P2X7 receptor activation, caspases activation, and chromatin condensation are differently affected by these three bisphenols, despite their structural homology. Previous studies comparing BPF and BPS were focused either on the quantification in the human placenta [42], on the potential transfer across the human placental barrier [44,45,46,47,48], or on their toxic effects in animal models [37,45,46,47,48]. None of them compared the effects of BPA, BPF, or BPS on human placental cell survival. It is of great importance to use human models to study placental toxicity, because the placenta is very different from species to species in terms of form, structure, and endocrine secretion [49].

BPA and BPF induced similar levels of P2X7 receptor activation, BPF being slightly more potent than BPA. BPS induced lower activation than both BPA and BPF, but is the only bisphenol that enhanced P2X7 receptor expression. The P2X7 receptor plays a key role in inflammation by NLRP3-inflammasome formation, leading to caspase-1 activation [50,51]. In our model, BPA and BPF significantly activated caspase-1, suggesting their implication in inflammasome induction. Prolonged activation of P2X7 receptor has been linked to apoptosis [52,53]. Depending on the cleaved caspase, apoptosis can be initiated via the extrinsic receptor mediated pathway through caspase-8 activation [54,55] or via the mitochondria-mediated pathway, resulting in caspase-9 activation [53]. Both pathways can be triggered by P2X7 receptor and lead to caspase-3 activation [56], followed by chromatin condensation [57]. Caspase-9 and caspase-3 were significantly activated by BPA and BPF, but only BPF enhanced caspase-8 activity. All tested bisphenols induced chromatin condensation. BPF seems to induce both extrinsic apoptosis mediated by caspase-8 and intrinsic apoptosis mediated by caspase-9, leading to caspase-3 activation and chromatin condensation. BPA only induces intrinsic apoptosis (caspase-9), also leading to caspase-3 activation and chromatin condensation. Contrary to BPA and BPF, BPS enhanced chromatin condensation without activation of caspase 8 or caspase 9, suggesting a caspase-independent mechanism [58].

## 5. Conclusions

**Scheme 1:**
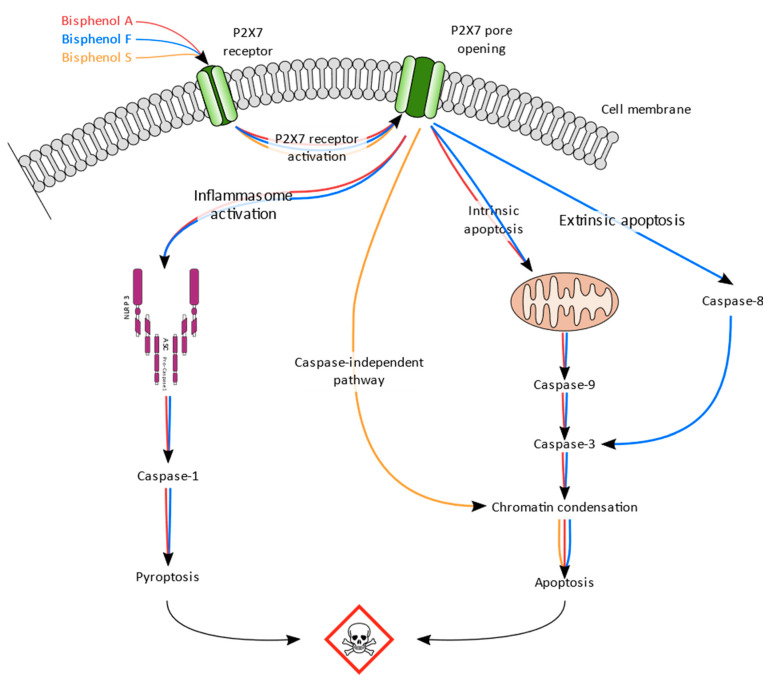
Synthesis scheme of the BPA, BPF, and BPS effects in human placental cells (JEG-Tox cells).

In conclusion, the results of our study suggest that BPA, BPF, and BPS induce toxicity in human placental cells. Despite their structural homology, the induced pathways are different, but they all share P2X7 receptor activation as the key starting event, reported to trigger preeclampsia in clinics (Scheme 1). BPF and BPS are therefore susceptible to inducing the same toxic effects in pregnant women, including preeclampsia, as BPA. BPA substitution by BPF and BPS is not safe for human health, particularly for pregnant women and their fetus.
